# Local anesthetic systemic toxicity induced by penile nerve block: A systematic review

**DOI:** 10.1097/MD.0000000000044582

**Published:** 2025-09-12

**Authors:** Miyeong Park, Soo Hee Lee, Ju-Tae Sohn

**Affiliations:** a Department of Anesthesiology and Pain Medicine, Gyeongsang National University Changwon Hospital 11, Samjeongja-ro, Seongsan-gu, Changwon-si, Gyeongsangnam-do, Republic of Korea; b Department of Anesthesiology and Pain Medicine, Gyeongsang National University College of Medicine, Jinju-si, Gyeongsangnam-do, Republic of Korea; c Department of Anesthesiology and Pain Medicine, Gyeongsang National University College of Medicine, Gyeongsang National University Hospital, Jinju-si, Gyeongsangnam-do, Republic of Korea; d Institute of Health Science, Gyeongsang National University, Jinju-si, Republic of Korea.

**Keywords:** bupivacaine, extreme age, inadvertently intravascular absorption, lidocaine, local anesthetic systemic toxicity, low muscle mass, overdose, penile nerve block, risk factor

## Abstract

**Background:**

Local anesthetics are widely used by non-anesthesiologists to provide analgesia. Penile nerve blocks are used for analgesia during urological surgery. Local anesthetic systemic toxicity (LAST), which is rare but likely fatal, is often caused by a penile nerve block. However, there has been no analysis of cases of LAST induced by a penile nerve block. This Preferred Reporting Items for Systematic Reviews and Meta-Analyses-compliant systematic review aimed to analyze case reports involving LAST induced by a penile nerve block, with a particular focus on the presumed causes, local anesthetics, and lipid emulsion treatment.

**Methods:**

Relevant case reports included in PubMed from inception to December 31, 2024, were retrieved using the keywords “penile nerve block,” “dorsal penile nerve block,” “penile block,” “local anesthetic toxicity,” “local anesthetic systemic toxicity,” and “local anesthetic overdose.”

**Results:**

Eleven case reports including 19 patients were obtained. The main presumed causes of penile nerve block-induced LAST were overdose (47.4%) and inadvertent intravascular absorption (47.4%) of local anesthetics. The main local anesthetics associated with overdose and inadvertent intravascular absorption were lidocaine (77.8%) and bupivacaine (88.9%), respectively. Of the patients, 89.5% had risk factors for LAST. Of the patients with LAST, 31.6% received lipid emulsion plus supportive treatment. The age distribution of patients with penile nerve block-induced LAST was as follows: <1 year (73.7%), ≥1 year to <19 years (15.8%), and ≥19 years (10.5%). A penile nerve block was used for circumcision in 84.2% of the cases. The most common symptoms of penile nerve block-induced LAST were cardiovascular symptoms (52.6%) and central nervous system symptoms (42.1%). The negative aspiration technique was used to prevent LAST in 47.4% of patients. All patients recovered from LAST.

**Conclusion:**

LAST in infants and neonates receiving a penile nerve block for circumcision is mainly caused by lidocaine overdose or inadvertent intravascular absorption of bupivacaine. The following measures should be considered to prevent LAST: adherence to the maximum recommended dose of local anesthetics; awareness of LAST risk factors; slow injection of the local anesthetic with minimal pressure and negative aspiration; and lipid emulsion preparation.

## 1. Introduction

Local anesthetics are widely used to provide analgesia for various procedures. However, inappropriate administration of local anesthetics can lead to local anesthetic systemic toxicity (LAST). LAST is a rare but often fatal condition caused by an excessive dose of a local anesthetic or its inadvertent intravascular absorption.^[1]^ The incidence of LAST under regional anesthesia performed by anesthesiologists, excluding spinal anesthesia, between 2011 and 2013 in Finnish anesthesia departments was reported to be 0.7 per 10,000.^[2]^ In addition, the incidence of LAST after peripheral nerve blocks in an Italian registry was reported to be approximately 0.34 per 1000 peripheral nerve blocks.^[3]^ Furthermore, the estimated incidence of LAST in children has been reported to be 8 per 100,000 blocks.^[4]^

LAST induces central nervous system symptoms, such as confusion, tinnitus, and seizures, followed by cardiovascular symptoms, including hypotension, cardiac arrhythmia, and cardiac arrest.^[5]^ Analysis of case reports and online registries regarding lipid emulsion treatment for LAST suggests that the penile nerve block (dorsal nerve block of the penis) is one of the most common nerve blocks associated with LAST.^[6]^ The incidence of LAST by block type is as follows: penile nerve block (23%), local tissue infiltration (17%), neuraxial block (13%), paravertebral block (8.5%), upper extremity block (8.5%), lower extremity block (8.5%), and head and neck block (8.5%).^[6]^ Penile and caudal nerve blocks are mainly involved in LAST cases reported in children.^[4]^ A penile nerve block is commonly used by surgeons for various urologic procedures, such as circumcision, penile debridement, corporal aspiration of priapism, and penile biopsy.^[7]^ In children, a penile nerve block, which is combined with light sedation, provides longer postoperative analgesia than general anesthesia.^[8]^ Thus, it is frequently administered for prolonged postoperative analgesia in children undergoing circumcision under general anesthesia. The penis contains highly vascularized tissue, and its dorsal nerve is located close to the dorsal artery and deep dorsal vein within the confined space of the penis.^[9]^ Hepatic microsomal enzymes contribute to the metabolism of amino-amide local anesthetics, which strongly bind to α_1_-acid glycoprotein.^[10]^ Infants and neonates, who undergo circumcision, have immature hepatic microsomal enzymes and low levels of the α_1_-acid glycoprotein. These factors contribute to the increased risk of LAST in this population.^[10]^ Given these physiological and anatomical factors, penile nerve blocks in children, especially during circumcision, represent a high-risk scenario for LAST. To mitigate this risk, prompt recognition and proper management of LAST are essential.

Lipid emulsions, first developed to meet nutritional requirements via parenteral administration, are now routinely used by anesthesiologists in the clinical management of systemic toxicity due to local anesthetic agents.^[1]^ Such treatment requires adequate knowledge regarding local anesthetics and lipid emulsion treatment. However, physicians other than anesthesiologists do not have sufficient knowledge of local anesthetics and lipid emulsion treatments for LAST.^[11,12]^ Additionally, a previous questionnaire-based study evaluating healthcare professionals’ knowledge of local anesthetic pharmacology, usage, and toxicity revealed that obstetrics and gynecology professionals had inadequate knowledge levels compared to anesthesiologists.^[13]^ Thus, there is concern about the preparedness of clinicians (outside of the anesthesiology specialty) regarding LAST when performing penile nerve blocks. To our knowledge, a systematic analysis of LAST cases caused by penile nerve blocks has not been reported.^[4,6–13]^ Thus, the goal of this systematic review was to analyze cases of LAST caused by penile nerve blocks, with a particular focus on the presumed cause, local anesthetics involved, risk factors for LAST, and lipid emulsion treatment. Based on the findings, preventive strategies were proposed.

## 2. Methods

**Figure 1.**
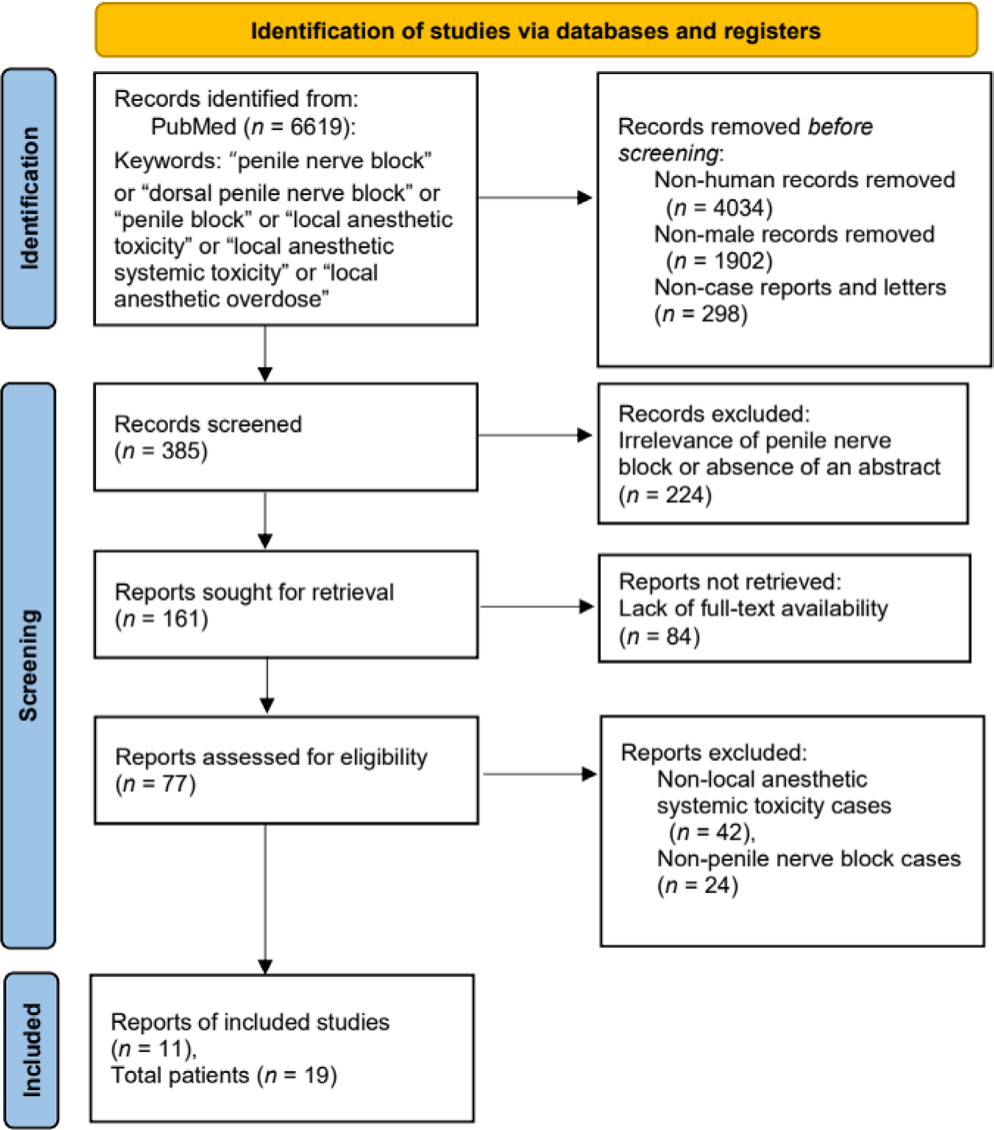
A total of 6619 articles were identified through PubMed searches using the following keywords: “penile nerve block” or “dorsal penile nerve block” or “penile block” or “local anesthetic toxicity” or “local anesthetic systemic toxicity” or “local anesthetic overdose.” Of these, 4043, 1902, and 298 articles were excluded because they were articles pertaining to nonhuman subjects, articles not involving human male patients, or articles other than case reports or letters, respectively. The titles and abstracts of the remaining 385 articles were screened for relevance to this review. Next, 224 articles were excluded because they did not contain an abstract or were unrelated to penile nerve block. The remaining 161 articles were further evaluated and 84 were removed because of a lack of full-text availability. The remaining 77 articles were assessed for eligibility. Of these, 66 articles were excluded for the following reasons: 42 articles did not report cases of local anesthetic systemic toxicity, and 24 articles did not involve penile nerve blocks. Finally, 11 articles met the inclusion criteria. Among these, one article reported 3 cases and another reported 7 cases, resulting in 19 patients who were included in this systematic review.

The requirement of approval from an institutional review board was waived because the study analyzed previously published case reports. Since this systematic review is based on previously published case reports of LAST induced by penile nerve block, patient consent is not required. A total of 6619 articles included in PubMed up to December 31, 2024, were retrieved using the following keywords: “penile nerve block” or “dorsal penile nerve block” or “penile block” or “local anesthetic toxicity” or “local anesthetic systemic toxicity” or “local anesthetic overdose” (Fig. 1). Further, 4043, 1902, and 298 articles were excluded because they were articles pertaining to nonhuman subjects, articles not involving human male patients, and articles other than case reports or letters, respectively. The titles and abstracts of the remaining 385 articles were screened for relevance to this review concerning case reports. Next, 224 of the 385 articles were excluded because they did not contain an abstract or were unrelated to penile nerve blocks. The remaining 161 articles were further evaluated and 84 were excluded because of a lack of full-text availability. The remaining 77 articles were assessed for eligibility. Of these, 42 and 24 articles were removed because they pertained to non-LAST cases and non-penile nerve blocks, respectively. Finally, 11 case reports were retrieved for analysis. One article included 3 case reports and another contained 7. Two authors (Park M and Lee SH) searched, reviewed, and verified cases of LAST induced by a penile nerve block. The following data were retrieved from each case report: publication year; patient age and weight; risk factors for LAST; purpose of using local anesthetics; whether general anesthesia was used; type of local anesthetic used; estimated dose of the local anesthetic used; onset and presumed cause of LAST; measures to prevent LAST; symptoms of LAST; lipid emulsion treatment; type of lipid emulsion; method of lipid emulsion administration; treatment; and outcome (Table 1).

**Table 1:** Overview of patient characteristics associated with local anesthetic systemic toxicity following a penile nerve block.

Case no.	Pub year	Age	Wt (kg)	Risk factors	Purpose to use LA	Under GA	LA	Estimated doses of LA	Onset of LAST	Presumed cause of LAST	Measures to prevent LAST	Sx of LAST	LE Tx	Type of LE	Method of LE Adm	Tx	Outcome
1^[14]^	2011	6 wk	3.2	Extreme age	Circumcision (pain control)	N	LDC	35 mg (10.9 mg/kg)	30 min	Overdose	N	GTC Sz	N			Supportive care, BDZ Mx	Recovery
2^[14]^	2011	23 d	3.5	Extreme age	Circumcision (pain control)	N	LDC	15 mg (4.2 mg/kg)	2 h	Overdose	N	Generalized Sz, apneic, cyanotic	N			Supportive care, BDZ Mx	Recovery
3^[14]^	2011	3 mo	NA	Extreme age	Circumcision (pain control)	N	LDC	6 mg/kg	50 min	Overdose	N	GTC Sz	N			Supportive care, BDZ Mx	Recovery
4^[15]^	2012	21 d	2.715	Extreme age, low body weight	Circumcision (pain control)	N	BPV	3.7 mg/kg	Within minutes	Overdose	N	Lethargy, disconjugate gaze, intermittent exotropia, altered consciousness, hypotonia	N			ICU care	Recovery
5^[16]^	2014	4 mo	8.9	Extreme age	Circumcision (pain control)	N	LDC	100 mg (11 mg/kg)	Immediately	Overdose	N	Abnormal behavior, GTC Sz Erythematous rash on his whole body, locked jaw	N			Supportive care	Recovery
6^[17]^	2014	7 wk	4.5	Extreme age	Circumcision (pain control)	N	LDC	30 mg (6.6 mg/kg)	20–30 min	Overdose	N	Generalized Sz, unresponsiveness, hypertonic	N			Supportive care, BDZ Mx	Recovery
7^[18]^	2014	11 mo	8	Extreme age	Circumcision (pain control)	Y	BPV	40 mg (5 mg/kg)	Shortly thereafter	Overdose	N	VT	Y	Intralipid 20%	Bolus + infusion	Supportive care	Recovery
8^[19]^	2015	4 mo	NA	Extreme age	Circumcision (pain control)	N	LDC	16 mg/kg	Immediately	Overdose	N	GTC Sz, VT	Y	Intralipid 20%	2 mL/kg	CPR, diazepam rectal injection, ECG monitoring, ICU care, brain W/U, blood test	Recovery
9^[20]^	2016	9 mo	10.4	Extreme age	Circumcision (pain control)	Y	BPV	25 mg (2.404 mg/kg)	15 min	IVA	NAT	Apnea, wide QRS, peaked T-wave	N			Supportive care	Recovery
10^[20]^	2016	20 mo	12.3	Extreme age	Circumcision (pain control)	Y	BPV	20 mg (1.626 mg/kg)	2–3 min	IVA	NAT	ST-segment depression, inverted T wave	N			Supportive care	recovery
11^[20]^	2016	14 mo	9.7	Extreme age	Distal hypospadias repair surgery (pain control)	Y	BPV	<5 mg (<0.515 mg/kg)	15 min	IVA	NAT	Wide QRS, peaked T-waves, hypotension	N			Supportive care	Recovery
12^[20]^	2016	9 mo	8.5	Extreme age	Circumcision (pain control)	Y	BPV	21.25 mg (2.5 mg/kg)	Immediately	IVA	NAT	Tachycardia, atrial flutter, hypotension	N			Supportive care	Recovery
13^[20]^	2016	10 mo	10.5	Extreme age	Distal hypospadias repair surgery (pain control)	Y	BPV	25 mg (2.381 mg/kg)	Immediately	IVA	NAT	Apnea, ST-segment depression, QRS widening	N			Adenosine	Recovery
14^[20]^	2016	6 mo	9.1	Extreme age	Circumcision (pain control)	Y	BPV	22.5 mg (2.473 mg/kg)	Immediately	IVA	NAT	Hypotension, bradycardia, ST-T change, peaked T-wave, QRS widening, peaked T-wave	Y	Intralipid 20%	Bolus	CPR	Recovery
15^[20]^	2016	6 mo	8.1	Extreme age	Circumcision (pain control)	Y	BPV	20 mg (2.469 mg/kg)	30 s	IVA	NAT	Apnea, bradycardia, hypotension, ST-T change, peaked T-wave	Y	Intralipid 20%	Bolus	CPR	Recovery
16^[21]^	2016	4.5 yr	26	Extreme age	Circumcision (pain control)	Y	RPV	50 mg (1.923 mg/kg)	2–3 min	IVA	NAT	Severe bradycardia, profound hypotension, ST depression, lethargy, extreme agitated, headache	N			Supportive care	Recovery
17^[22]^	2018	54 yr	112	X	Circumcision (pain control)	Y	BPV	80 mg (0.714 mg/kg)	13 min	IVA	NAT	Tachycardia with multiple VPC, broad QRS complexes with occasional VPC	Y	20% LE	Bolus + infusion	Supportive care	Recovery
18^[23]^	2024	39 yr	NA	X	Corporal aspiration	N	LDC	2000 mg	NA	Overdose	N	Hallucination, visual loss, agitation	Y	20% LE	Infusion	Supportive care, ICU care	Recovery
19^[24]^	2025	12 wk	NA	Extreme age	Circumcision (pain control)	N	LDC	NA	5 min	UD	N	GTC Sz	N			Supportive care	Recovery

Adm = administration, BDZ = benzodiazepine, BPV = bupivacaine, CPR = cardiopulmonary resuscitation, d = days, ECG = electrocardiogram, GA = general anesthesia, GTC = generalized tonic clonic, hr = hour, ICU = intensive care unit, IVA = inadvertently intravascular absorption, LA = local anesthetic, LAST = local anesthetic systemic toxicity, LDC = lidocaine, LE = lipid emulsion, min = minutes, mo = months, Mx = medication, N = none, NA = not available, NAT = negative aspiration technique, Pub = publication, RPV = ropivacaine, s = second, Sx = symptom, Sz = seizure, Tx = treatment, UD = undetermined, VPC = ventricular premature contraction, VT = ventricular tachycardia, W/U = workup, wk = weeks, Wt = weight, X = none, Y = yes, yr = year.

In this analysis, if the dose of the local anesthetic used in the penile nerve block exceeded the maximum recommended amount, the presumed cause of LAST was considered to be an overdose of the local anesthetic. Conversely, if the dose was below the maximum recommended level, the presumed cause was inadvertent intravascular absorption of the local anesthetic. However, if the dose of the local anesthetic used in the penile nerve block was not available, the presumed cause of LAST was considered “undetermined.” In addition, this analysis considered the risk factors for LAST, including extreme age (age < 16 or ≥60 years), low muscle mass (in infants and neonates), and comorbidities.^[25]^ Thus, the presumed causes of LAST in the current analysis were as follows: overdose and risk factors, overdose alone, inadvertent intravascular absorption and risk factors, inadvertent intravascular absorption alone, undetermined cause and risk factor, and undetermined alone. Each parameter to be analyzed was expressed as a percentage of the total number of patients (N = 19, or the total number of patients in each group). Owing to the rounding up of individual item percentages, the cumulative total may not precisely sum up to 100%.

## 3. Results

Eleven case reports with LAST induced by a penile nerve block, which contained 19 patients, were retrieved from PubMed (Table 1 and Fig. 1).^[14–24]^ N indicates the number of patients.

### 3.1. Age distribution of patients with LAST induced by a penile nerve block

**Figure 2.**
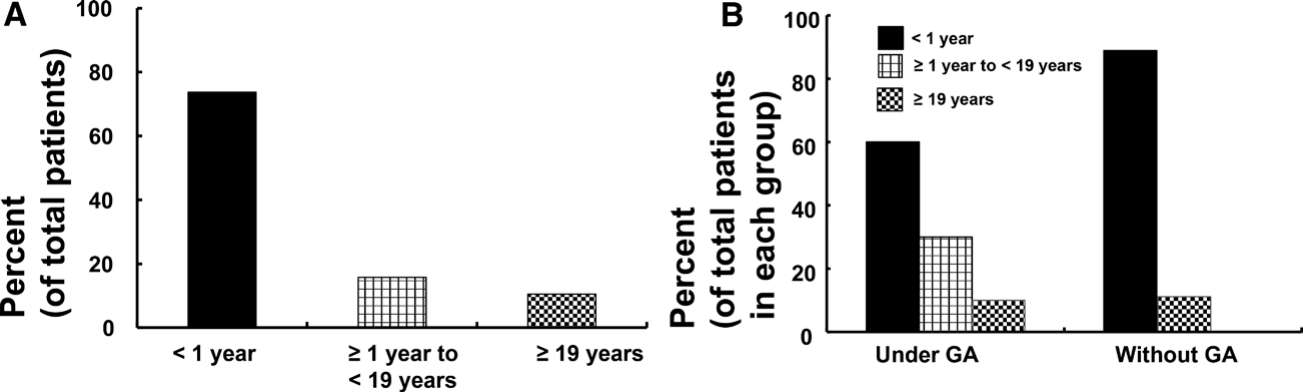
(A) Age distribution of patients (total number of patients: 19) with local anesthetic systemic toxicity (LAST) induced by a penile nerve block. (B) Age distribution of patients with LAST induced by a penile nerve block with (total number of patients: 10) or without (total number of patients: 9) general anesthesia (GA).

The age distribution of patients with LAST due to a penile nerve block was as follows: <1 year, 73.7% (N = 14); ≥1 year to <19 years, 15.8% (N = 3); and ≥19 years, 10.5% (N = 2) (Table 1, Fig. 2A).

### 3.2. Purpose of penile nerve block use in patients with LAST induced by a penile nerve block

The purposes of using a penile nerve block, which was associated with LAST, were as follows: circumcision (84.2%, N = 16), distal hypospadias repair (10.5%, N = 2), and corporal aspiration for priapism (5.3%, N = 1) (Table 1).

### 3.3. Local anesthetics associated with LAST induced by a penile nerve block

**Figure 3.**
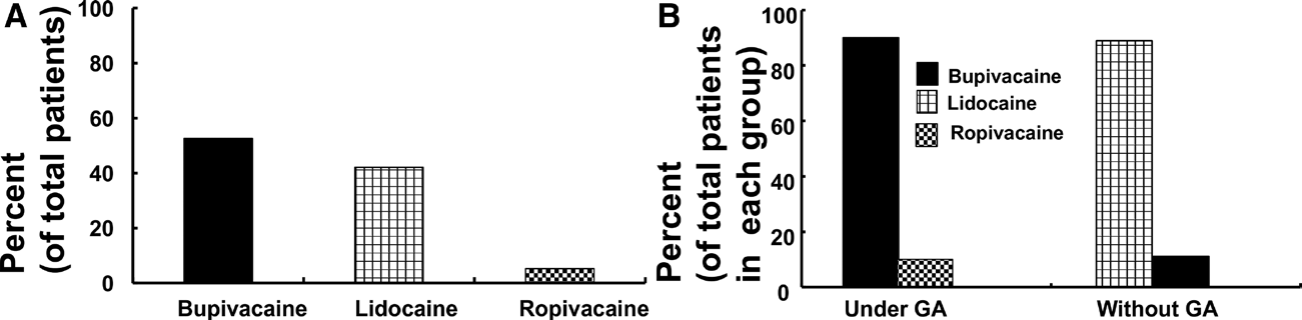
(A) The types of local anesthetics (total number of patients: 19) involved in local anesthetic systemic toxicity (LAST) induced by a penile nerve block. (B) The types of local anesthetics involved in LAST induced by a penile nerve block with (total number of patients: 10) or without (total number of patients: 9) general anesthesia (GA).

The types of local anesthetics used in the penile nerve block associated with LAST were as follows: bupivacaine (52.6%, N = 10), lidocaine (42.1%, N = 8), and ropivacaine (5.3%, N = 1) (Table 1, Fig. 3A). The local anesthetics (N = 10) used in the penile nerve block associated with LAST under general anesthesia were bupivacaine (90%, N = 9) and ropivacaine (10%, N = 1) (Fig. 3B). The local anesthetics (N = 9) used in the penile nerve block associated with LAST without general anesthesia were lidocaine (88.9%, N = 8) and bupivacaine (11.1%, N = 1) (Fig. 3B).

### 3.4. Presumed causes of LAST induced by a penile nerve block and associated risk factors

**Figure 4.**
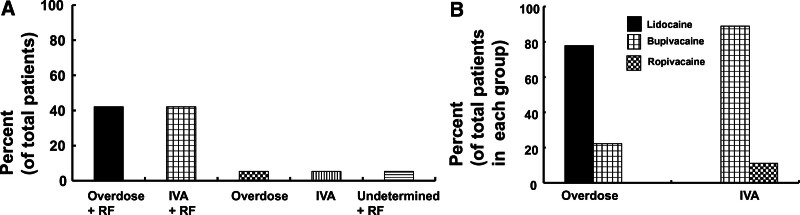
(A) Frequency of the presumed cause with or without risk factors (RF) for local anesthetic systemic toxicity (LAST) in the patients (total number of patients: 19) with LAST induced by a penile nerve block. IVA = inadvertent intravascular absorption. (B) Frequency of local anesthetics involved in overdose (total number of patients: 9) or IVA (total number of patients: 9) of local anesthetics as a presumed cause of LAST. LAST = local anesthetic systemic toxicity.

The presumed causes of LAST induced by penile nerve block were overdose (47.4%, N = 9), inadvertent intravascular absorption (47.4%, N = 9), and undetermined (5.3%, N = 1) (Table 1). Of all the patients, 89.5% (N = 17) had risk factors for LAST, such as extreme age or low muscle mass while 10.5% (N = 2) did not (Table 1). Considering the risk factors for LAST, the frequency of the presumed causes of LAST was as follows (Table 1 and Fig. 4A): local anesthetic overdose and risk factors (42.1%, N = 8), inadvertent intravascular absorption of the local anesthetic and risk factors (42.1%, N = 8), overdose alone (5.3%, N = 1), inadvertent intravascular absorption alone (5.3%, N = 1), or undetermined cause and risk factors (5.3%, N = 1). The local anesthetics associated with overdose (total N = 9) as the presumed cause of LAST were lidocaine (77.8%, N = 7, 9.12 ± 4.34 [mean ± SD] mg/kg) and bupivacaine (22.2%, N = 2; median: 4.35 mg/kg [Q1–Q3: 4.025–4.675 mg/kg]) (Table 1 and Fig. 4B). The local anesthetics associated with inadvertent intravascular absorption (N = 9) as the presumed cause of LAST were bupivacaine (88.9%, N = 8) and ropivacaine (11.1%, N = 1). The local anesthetic associated with an undetermined presumed cause of LAST was lidocaine (N = 1).

### 3.5. Frequency of LAST-associated penile nerve blocks performed under general anesthesia

Of the patients, 52.6% (N = 10) received a penile nerve block under general anesthesia, while 47.4% (N = 9) received it without general anesthesia (Table 1). The age distribution of the patients (N = 10) who received a penile nerve block associated with LAST under general anesthesia was as follows: <1 year (60%, N = 6), ≥1 year to <19 years (30%, N = 3), and ≥19 years (10%, N = 1) (Table 1 and Fig. 2B). The age distribution of the patients (N = 9) who received a penile nerve block associated with LAST without general anesthesia was <1 year (88.9%, N = 8) and ≥19 years (11.1%, N = 1) (Table 1 and Fig. 2B).

### 3.6. Measures to prevent LAST in patients with LAST induced by a penile nerve block

No measures to prevent LAST due to a penile nerve block were used in 52.6% (N = 10) of the patients, and only the negative aspiration technique was used during the penile nerve block in 47.4% (N = 9) of the patients (Table 1).

### 3.7. Symptoms of LAST induced by a penile nerve block

**Figure 5.**
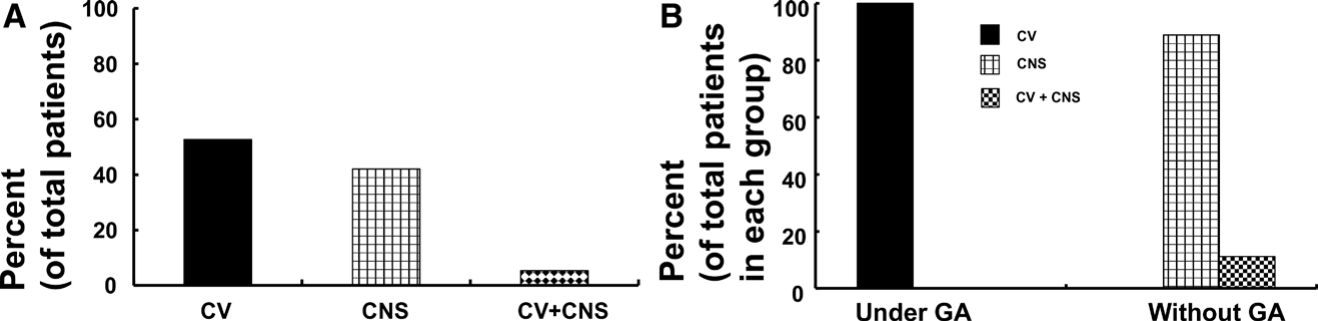
(A) Frequency of symptoms in patients (total number of patients: 19) with local anesthetic systemic toxicity (LAST) induced by a penile nerve block. CNS = central nervous system symptoms; CV = cardiovascular symptom; CV + CNS = cardiovascular symptoms plus central nervous systemic symptoms. (B) Frequency of symptoms in patients with LAST induced by a penile nerve block with (total number of patients: 10) or without (total number of patients: 9) general anesthesia (GA).

The frequencies of symptoms due to LAST induced by a penile nerve block were as follows: cardiovascular symptoms (52.6%, N = 10), symptoms involving the central nervous system (42.1%, N = 8), and symptoms involving the cardiovascular and central nervous systems (5.3%, N = 1) (Table 1 and Fig. 5A). The symptoms of LAST caused by a penile nerve block under general anesthesia were 100% (N = 10) cardiovascular in nature (Table 1 and Fig. 5B). However, the symptoms of LAST caused by a penile nerve block without general anesthesia were as follows: central nervous system symptoms (88.9%, N = 8) and symptoms involving the cardiovascular and central nervous systems (11.1%, N = 1) (Table 1 and Fig. 5B).

### 3.8. Treatments for LAST induced by a penile nerve block

The following supportive treatments were used in patients with LAST induced by a penile nerve block: oxygenation, 100% oxygen, intubation, midazolam, sodium thiopental, diazepam, intensive care unit observation, cardiopulmonary resuscitation, and atropine (Table 1).

### 3.9. Lipid emulsion treatment for LAST induced by a penile nerve block

**Figure 6.**
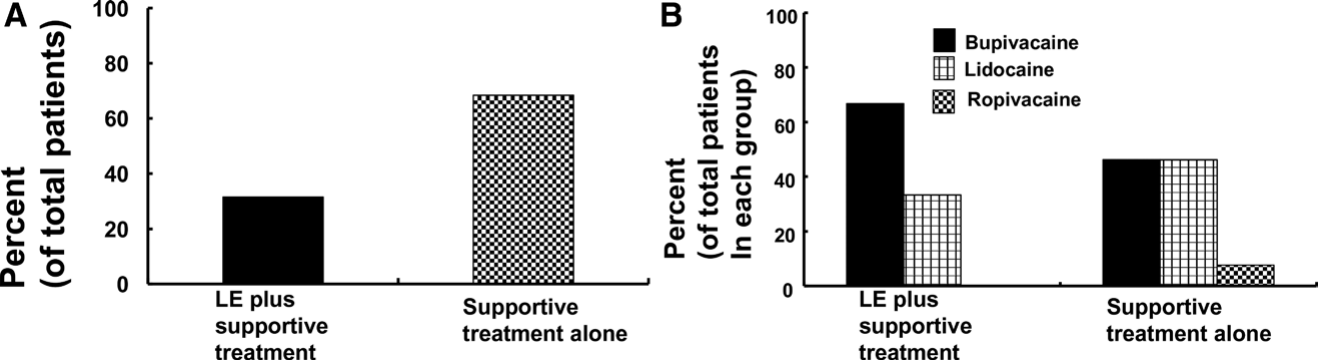
(A) Frequency of lipid emulsion (LE) treatment with supportive treatment, and supportive treatment alone in patients (total number of patients: 19) with local anesthetic systemic toxicity (LAST) induced by a penile nerve block. (B) Frequency of local anesthetics involved in LAST induced by a penile nerve block requiring lipid emulsion therapy plus supportive treatment (total number of patients: 6) versus supportive treatment alone (total number of patients: 13).

Lipid emulsion treatment for LAST induced by a penile nerve block in an 11-month-old infant was first reported in 2014 (Table 1, Case no. 7).^[18]^ Lipid emulsion treatment was used in 31.6% of the total patients (N = 19) along with supportive treatment (Case nos. 7, 8, 14, 15, 17, and 18; Fig. 6A). The remaining 68.4% (N = 13) of the patients received only supportive treatment without lipid emulsion treatment (Table 1; Fig. 6A). Among the lipid emulsions used, intralipid was used in 66.7% (N = 4) of the patients (total number of patients: 6) receiving lipid emulsion treatment. However, only 20% lipid emulsions were described in 33.3% (N = 2) of the patients receiving lipid emulsion treatment. The lipid emulsion administration methods used were as follows: bolus administration alone (50%, N = 3), bolus administration followed by continuous infusion (33.3%, N = 2), and infusion alone (16.7%, N = 1) (Table 1). The persons who recommended lipid emulsion treatment for LAST were as follows: anesthesiologists (N = 2, 33.3%) and emergency department staff (N = 1, 16.7%). Information regarding physicians recommending lipid emulsion treatment was not available for 3 cases (50%, N = 3). Four patients (66.7% of the total number of patients who received lipid emulsion treatment) with LAST due to a penile nerve block received lipid emulsion treatment under general anesthesia (Table 1), whereas 2 with LAST caused by a penile nerve block received lipid emulsion treatment without general anesthesia (Table 1). The local anesthetics involved in LAST induced by a penile nerve block for which lipid emulsion treatment was needed along with supportive treatment (total number of patients: 6) were bupivacaine (66.7%, N = 4) and lidocaine (33.3%, N = 2) (Table 1, Fig. 6B). Further, the local anesthetics involved in LAST induced by a penile nerve block for which supportive treatment alone was necessary were bupivacaine (46.2%, N = 6), lidocaine (46.2%, N = 6), and ropivacaine (7.7%, N = 1) (total number of patients: 13) (Table 1, Fig. 6B).

### 3.10. Outcomes in patients with LAST induced by a penile nerve block

All patients recovered from LAST resulting from a penile nerve block, regardless of whether they received supportive care alone (68.4 %, N = 13) or in combination with lipid emulsion treatment (31.6%, N = 6) (Table 1).

## 4. Discussion

In this study, we systematically reviewed case reports involving LAST induced by a penile nerve block, with a particular focus on the presumed causes, local anesthetics, and lipid emulsion treatment. Our findings suggest that an overdose of lidocaine or inadvertent intravascular absorption of bupivacaine induces LAST due to a penile block in infants and neonates who undergo circumcision. The main findings of this systematic review are as follows. LAST induced by a penile nerve block developed most commonly in neonates and infants (73.7%). Overdose of lidocaine (77.8% of overdose) or inadvertent intravascular absorption of bupivacaine (88.9% of inadvertent intravascular absorption) was commonly involved in LAST induced by a penile nerve block. LAST due to a penile nerve block developed frequently in children who undergo circumcision (84.2%). Finally, 31.6% of all patients with LAST induced by a penile nerve block received supportive treatment plus lipid emulsion treatment, whereas 68.4% received supportive treatment alone.

The maximum recommended dosages of local anesthetics without epinephrine are as follows^[5]^: lidocaine, 3 mg/kg; bupivacaine, 2 mg/kg; and ropivacaine, 3 mg/kg. The mean lidocaine overdose and median bupivacaine overdose observed in this analysis were as follows. Overdoses of lidocaine (N = 6, 9.12 mg/kg) and bupivacaine (N = 2, 4.35 mg/kg) exceeded the maximum recommended doses of lidocaine and bupivacaine by approximately 3.04 and 2.18 times, respectively. Hepatic microsomal enzymes contribute to the metabolism of amino-amide local anesthetics, and α_1_-acid glycoprotein binds to amino-amide local anesthetics.^[10]^ In addition, infants and neonates, who accounted for 73.7% of patients with LAST induced by a penile nerve block in the current analysis, have immature hepatic function and a low α_1_-acid glycoprotein level. Thus, these factors may have additively contributed to LAST induced by a penile nerve block owing to an overdose or inadvertent absorption of the local anesthetic. Moreover, the dorsal nerve of the penis lies close to the dorsal artery and deep dorsal vein of the penis.^[9]^ These structures (the dorsal nerve, dorsal artery, and deep dorsal vein of the penis) are situated in a small compartment between Buck’s fascia and the tunica albuginea.^[9]^ Thus, this factor may also contribute to the inadvertent intravascular absorption of local anesthetics owing to unrecognized high pressure during local anesthetic administration. Furthermore, low muscle mass in infants, which was observed in the current analysis, may contribute to a high risk of LAST.^[25]^ As found in a previous report, LAST was most frequently observed in neonates and infants younger than 1 year of age in the current analysis.^[4]^ Taken together, the study findings suggest that the maximum recommended dose of local anesthetic should be adhered to, and risk factors for LAST, which include extreme age, immature hepatic function, low muscle mass, and close location of the dorsal nerve and vessel of the penis, should be considered to prevent LAST induced by the penile nerve block used for circumcision in infants and neonates. If possible, bupivacaine should be avoided because it is highly cardiotoxic compared with other amino-amide local anesthetics.^[26]^ However, in the current analysis, we found that bupivacaine was commonly used to perform penile nerve blocks under general anesthesia (Fig. 3B), which may be attributed to the secure airway management provided by general anesthesia.

The cardiovascular symptoms observed in the current analysis included profound hypotension, severe bradycardia, and abnormal findings on electrocardiography, such as QRS widening, ST-segment depression, and an inverted T wave (Table 1). The central nervous system symptoms observed in this analysis included hallucinations, visual loss, status epilepticus, lethargy, seizure, altered consciousness, and unresponsiveness (Table 1). LAST usually produces central nervous system symptoms, followed by cardiovascular symptoms.^[5]^ However, in the current analysis, cardiovascular symptoms were more frequent compared with central nervous system symptoms. This may be because the penile nerve block was performed more frequently under general anesthesia (52.6%) than without general anesthesia (47.4%) in the cases analyzed in this study (Table 1). Thus, central nervous system symptoms such as seizures and agitation may have been masked by general anesthesia. Consistent with the results of the current analysis, a previous study reported that cardiovascular symptoms induced by LAST in children were most common under general anesthesia.^[4]^

The treatment of LAST involves several critical steps.^[1,25]^ First and foremost, securing the airway is essential for avoiding hypoxia, acidosis, and hypercapnia, all of which can exacerbate the condition. Second, seizures must be promptly managed with benzodiazepines, lipid emulsion treatment, and low-dose succinylcholine. Third, in cases of cardiac arrest, a reduced dose of epinephrine (<1 μg/kg) should be administered along with amiodarone and lipid emulsion, and advanced cardiac life support. In patients who do not respond to these interventions, cardiopulmonary bypass may be a last resort. The supportive treatments used for LAST induced by a penile nerve block in the current analysis included midazolam, oxygenation, intubation, adenosine, sodium thiopental, atropine, intensive care unit treatment, and close monitoring (Table 1).

The dose of the lipid emulsion treatment for LAST recommended by the American Society of Regional Anesthesia and Pain Medicine is as follows: 20% lipid emulsion 1.5 mL/kg bolus administration followed by 20% lipid emulsion continuous infusion at 0.25 mL/kg/min.^[25]^ In the current analysis, lipid emulsion was used in 4 cases of bupivacaine-induced LAST, which were due to bupivacaine overdose (1 case) or inadvertent intravascular absorption (3 cases), and in 2 cases of lidocaine-induced LAST, which were due to lidocaine overdose (Table 1 and Fig. 6B). The underlying mechanism of the lipid emulsion treatment for LAST involves both indirect and direct effects.^[1]^ The theory of lipid shuttle, which is an indirect effect, suggests that a lipid compartment created by lipid emulsion within the bloodstream absorbs highly lipid-soluble local anesthetics (drugs with a log *P* [octanol/water partition coefficient] > 2; log *P* of bupivacaine, ropivacaine, and lidocaine: 3.41, 2.9, and 2.44, respectively) from the heart and the brain.^[1]^ Then, the lipid emulsion containing the local anesthetic is transported to the liver, muscle, and adipose tissue for detoxification and storage.^[1]^ Thus, the frequent use of lipid emulsions for treating LAST induced by bupivacaine in the current analysis may be associated with the lipid solubility of bupivacaine being the highest (log *P*: 3.41) among the amino-amide local anesthetics and its relatively high cardiotoxic effect.^[1]^ In addition, lipid emulsion therapy has multiple direct physiological effects, such as enhancement of cardiac contractility, mitigation of mitochondrial impairment, supply of essential fatty acids, promotion of the phosphorylation of glycogen synthase kinase-3β, and suppression of the release of nitric oxide.^[1]^ Thus, lipid emulsions produce both scavenging of bupivacaine or lidocaine from the heart and brain via lipid shuttles and direct inotropic effects, which may contribute to recovery from LAST induced by a penile nerve block with bupivacaine or lidocaine. The doses of 20% lipid emulsion injected via bolus administration in the current analysis were 1.5 mL/kg (2 cases) and 2 mL/kg (1 case). The doses of lipid emulsion used in bolus administration followed by continuous infusion in the current analysis were 1.5 ml/kg followed by 0.25 mL/kg/min or 100 mL/122 kg followed by 200 mL/112 kg/20 min (0.089 mL/kg/min) and subsequently 0.5 mL/kg/min. The doses of lipid emulsion, such as 1.5 mL/kg alone (2 cases) or 1.5 mL/kg followed by 0.25 mL/kg/min (1 case), observed in this analysis, seem to be associated with the lipid emulsion doses for LAST recommended by the American Society of Regional Anesthesia and Pain Medicine.^[25]^ The dose of lipid emulsion administered via only continuous infusion in the current analysis was 840 mL/60 min (body weight: not available). Lipid emulsion therapy may lead to several complications including fat overload syndrome, pyrogenic responses, interference with diagnostic laboratory assessments, pancreatitis, and acute respiratory distress syndrome.^[27]^ However, in the present analysis, none of the 6 patients who received lipid emulsion treatment experienced any adverse effects attributable to the treatment. In the current analysis, 73.7% of the penile nerve blocks associated with LAST were performed by non-anesthesiologists, such as general practitioners or surgeons. However, information regarding physicians performing penile nerve block was not available for 26.3% of patients with LAST induced by a penile nerve block. Lipid emulsion treatment for LAST resulting from a penile nerve block was recommended by an anesthesiologist in 2 cases and by emergency medicine staff in another case. The first lipid emulsion treatment for LAST attributable to non-anesthesiologists (cardiologists) was reported in 2011.^[28]^ Further, the first lipid emulsion treatment for LAST induced by a penile nerve block in the current analysis was reported in 2014 (Case no. 7).^[18]^ Lipid emulsion was administered under less serious conditions in the current analysis, including cases with LAST induced only by penile nerve block, compared with intractable lidocaine toxicity attributable to the involvement of non-anesthesiologists.^[18–20,22,23,28]^ This difference in patient conditions for which lipid emulsion treatment was administered between the current analysis and previous case report may be due to the following factors: early lipid emulsion treatment for LAST has gained wide acceptance over time; of the patients who experienced LAST following a penile nerve block, 52.6% had a secured airway as a result of general anesthesia; and under general anesthesia, continuous monitoring using various surveillance devices, including electrocardiography and blood pressure measurement, facilitated the early detection of LAST signs and enabled prompt intervention. All the patients recovered from LAST in current analysis. Because lipid emulsion therapy is recommended for LAST, lipid emulsions should be prepared during a penile nerve block to treat unexpected LAST.

To prevent LAST due to a penile nerve block, a negative aspiration technique was used in 47.4% of the patients in the current analysis. A previous study suggested that although 52% of children with LAST undergo the negative aspiration technique, this technique does not reliably prevent LAST.^[29]^ Similar to a previous report, the current analysis showed that the negative aspiration technique used during penile nerve block did not reliably prevent LAST.^[29]^ Ultrasound-guided nerve block reduces the risk of LAST and postoperative neurological symptoms.^[30]^ A previous study reported that ultrasound- and landmark-guided penile nerve blocks performed by an experienced investigator in pediatric patients under general anesthesia did not produce adverse effects.^[31]^ This previous study had the following limitations in terms of comparison of LAST by landmark- and ultrasound-guided penile nerve block: the performance of penile nerve block by one experienced investigator, and a relatively small sample size (310 prepubertal patients).^[31]^ On the other hand, in the comparison of ultrasound-guided penile nerve block with spontaneous breathing and landmark-guided penile nerve block under general anesthesia, the ultrasound-guided penile nerve block was found to result in a shorter time to discharge from the end of surgery to the recovery room, lower opioid consumption, and lower pain level after surgery.^[32]^ By enabling real-time visualization of the penile neurovascular anatomy, the ultrasound-guided penile nerve block facilitates precise needle placement, minimizes the required anesthetic dose, and ensures effective local anesthetic dispersion, which may contribute to the reduced risk of LAST.^[33]^ However, a multi-institutional study is needed to further compare LAST induced by landmark-guided versus ultrasound-guided penile nerve blocks.

The current analysis has some limitations. First, LAST induced by a penile nerve block is usually less frequently reported than its actual incidence because of medicolegal problems, surgeons’ limited awareness of LAST, and atypical symptoms. Second, one case report included in the current analysis contained 7 patients, which accounted for 36.8% of total patients.^[20]^ Third, some information was unavailable for each of the cases analyzed. Thus, these factors may have affected the results of the current analysis. Therefore, further research using a standardized online registry and uniform reporting form for LAST induced by penile nerve block is needed to address these limitations. However, despite these limitations, we believe that this analysis contributes to the investigation of factors associated with LAST induced by penile nerve blocks and could help prevent LAST by promoting a better understanding of these factors.

Collectively, the results of our analysis suggest that an overdose of lidocaine or an inadvertent intravascular injection of bupivacaine causes LAST induced by a penile nerve block in infants and neonates who undergo circumcision. To prevent LAST associated with penile nerve blocks, the following measures should be considered: adherence to the maximum recommended doses of local anesthetics; awareness of increased risk factors for LAST in infants and children, such as immature hepatic microsomal enzyme activity, low levels of α_1_-acid glycoprotein, and low muscle mass; slow injection of local anesthetics with minimal pressure and frequent negative aspiration because of close proximity of the penile nerve and vessels; availability of lipid emulsion for immediate use, and use of ultrasound-guided penile nerve blocks.

## Author contributions

**Conceptualization:** Miyeong Park, Soo Hee Lee, Ju-Tae Sohn.

**Data curation:** Miyeong Park, Soo Hee Lee.

**Formal analysis:** Miyeong Park, Soo Hee Lee, Ju-Tae Sohn.

**Funding acquisition:** Ju-Tae Sohn.

**Investigation:** Miyeong Park, Soo Hee Lee, Ju-Tae Sohn.

**Methodology:** Miyeong Park, Soo Hee Lee, Ju-Tae Sohn.

**Project administration:** Ju-Tae Sohn.

**Resources:** Miyeong Park, Soo Hee Lee, Ju-Tae Sohn.

**Software:** Miyeong Park, Soo Hee Lee, Ju-Tae Sohn.

**Supervision:** Ju-Tae Sohn.

**Validation:** Miyeong Park, Soo Hee Lee, Ju-Tae Sohn.

**Visualization:** Miyeong Park, Soo Hee Lee, Ju-Tae Sohn.

**Writing – review & editing:** Miyeong Park, Soo Hee Lee, Ju-Tae Sohn.

**Writing – original draft:** Ju-Tae Sohn.
